# Metastatic renal cell carcinoma initially presented with an intramedullary spinal cord lesion: a case report

**DOI:** 10.4076/1757-1626-2-7805

**Published:** 2009-09-11

**Authors:** Mehrnaz Asadi, Hadi Rokni-Yazdi, Farahnaz Salehinia, Farshad S Allameh

**Affiliations:** 1 Department of General Internal Medicine, Imam Khomeini HospitalKeshavarz Boulevard, Medical Sciences/Tehran University, TehranIran; 2 Department of Radiology and Imaging, Imam Khomeini HospitalKeshavarz Boulevard, Medical Sciences/Tehran University, TehranIran; 3 Department of General Internal Medicine, Imam Khomeini HospitalKeshavarz Boulevard, Medical Sciences/Tehran University, TehranIran

## Abstract

**Introduction:**

One of the rare manifestations of systemic neoplasia is intramedullary spinal cord metastasis that causes serious diagnostic and therapeutic dilemma. It has been very rarely reported as the initial manifestation of carcinoma. This is report of a metastatic renal cell carcinoma initially presented with intramedullary spinal cord lesion, to our knowledge there are few similar reports in literature.

**Case presentation:**

We report a 51-year-old Iranian woman who presented with back pain and paraparesis. MR imaging study of her spine showed an enhancing cystic lesion at the level of conus medullaris. Despite detailed investigation, no specific aetiology was found till a bone scan obtained to evaluate an agonizing pain on the dorsum of the left hand revealed photon deficient area within the left kidney in addition to oseoblastic bony lesions. After thorough imaging investigation she underwent radical nephrectomy which confirmed renal cell carcinoma.

**Conclusion:**

Considering the prevalence of cancer, it is imperative that clinicians be mindful of occult carcinoma as the cause of suspicious intramedullary spinal cord lesion.

## Introduction

Carcinoma metastases of the spinal cord are rare diseases; the least frequent of which, is intramedullay spinal cod metastasis (ISCM) that causes serious diagnostic and therapeutic problems [1,2], however, despite it’s rarity, intramedullary metastasis should be considered in patients with systemic malignancy presenting with myelopathic symptoms [3]. The recognition of intrameullary spinal cord metastasis is an ominous finding as it generally occurs in the setting of widespread systemic and intracranial disease and is the prelude to cancer death by a few months [4].

The presenting symptoms of ISCM vary from pain, sensory loss, weakness, urinary incontinence to pseudo Brown-Sequard and/or Brown-Sequard syndrome [5]. The duration of symptoms before diagnosis of ISCM ranges from days to a few months [5,6]. The very rare occurrence of ISCM and the absence of pathognomic symptoms often lead to an undue delay until the underlying malignancy is discovered [7]. Considering the widely disseminated nature of the underlying malignancy at the time of diagnosis, ISCM is generally associated with poor survival and portends itself as a dismal finding within the context of a systemic cancer; heralding cancer death by a few months [6,8,9]. Surgery and radiotherapy have been recommended controversially in the treatment of ISCM. Although long-term survival is poor, treatment may preserve ambulation in the case of early diagnosis; it may also stabilize neurological function and this may change the patient’s health related quality of life unbelievably [5,7,11]. Heightened awareness of this entity may lead to early diagnosis at a stage when neurological deficits may be reversible and more effective palliation may be expected.

## Case presentation

**Figure 1.**
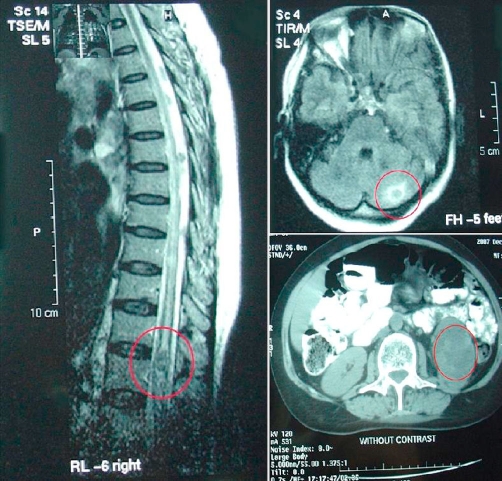
MR imaging of the spine at the level of conus medullaris. A non enhancing cystic lesion is seen in the area.

**Figure 2.**
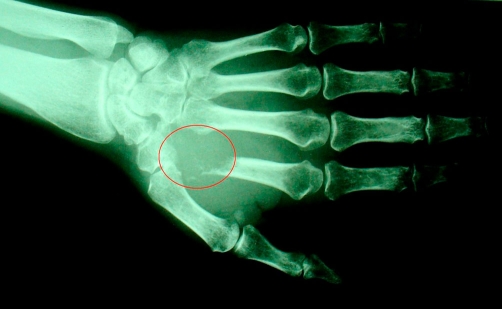
X-Ray imaging of the left hand showing osteolytic lesion of the second metacarpal bone.

**Figure 3.**
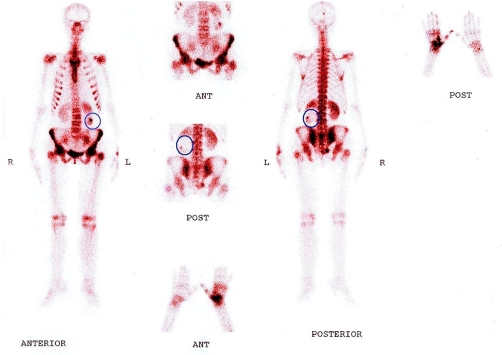
Whole body bone scan with 99 m Tc DTPA showing increased skeletal uptake of the radiotracer in several areas and photopenic area in the left kidney.

A 51-year-old Iranian woman presented with 2-month history of progressive unremitting back pain, exacerbated at night, superimposed on a creeping paraparesis. She also suffered from episodes of urinary retention for which she had undergone thorough clinical examination and a series of investigation including neuroimaging, cerebrospinal fluid analysis and also serologic surveys to find the aetiology. MR imaging of the brain and spine revealed one non-enhancing cystic lesion without surrounding enhancement located within the left cerebral hemisphere and another one at the level of conus medullaris in the spine (Figure 1). No infectious aetiology had been found despite performing detailed investigation to detect any sign of cysticercosis, brucellosis and/or HTLV infection. The tissue biopsy of the involved area was not obtained as she had not consented to it. An extremely painful induration with indistinct border was found over the dorsum of the left hand. X-Ray imaging showed osteolysis of the second metacarpal bone (Figure 2). The other sites of body skeleton were normal on examination despite her complaint about the agonizing pain over most parts of her body. 99 m Tc-DTPA revealed increased uptake of radiotracer in axial skeleton and left hand area and, also a photon-deficient area adjacent to the lower pole of the left kidney suggestive of a space occupying lesion originating from that kidney (Figure 3).

Complex cystic renal mass lesion was revealed on ultrasonography which was found to be hypervascular on contrast-enhanced CT scan. The result of imaging investigations was compatible with renal cell carcinoma diagnosis, till otherwise proved. In the meantime, the patient was hospitalized for nutritional supplementation and other supportive care. Radical nephrectomy was done afterwards and the histology confirmed renal cell carcinoma with sarcomatous component. Early in postoperative period she developed .thromboses in proximal veins of left lower extremity for which she received enoxaparin 60 mg twice daily subcutaneousely. She also had severe hypercalcemia probably due to paraneoplastic effect of the tumour producing PTHrP, and widespread bony metastases as the intact PTH assay was undetectable. She received appropriate treatment for her hypercalcemia. The patient and her family refused to accept further treatment when they were informed regarding the natural course and poor prognosis of the illness and the limitations of the currently available treatment, and she was lost for further follow up.

## Discussion

Spinal metastases may be seen in as much as 70% of patients with systemic neoplasia. Among these lesions, intramedullary spinal metastases are rare, comprising only 0.5% of spinal axis metastases. Majority of them arise from the lung neoplasia ,with small cell carcinoma being the predominant histological subtype. Breast, colorectal, renal, melanoma, thyroid and lymphoma have all infrequently been reported to be the origin. The lesions are found most often in patients with concomitant brain metastasis [12]. However, metastasis to the intramedullary spinal cord is extremely rare as the initial sign of a disseminated carcinoma [9]. The lesions are believed to result from leptomeningeal carcinomatosis with subsequent cerebrospinal fluid spread or as a result of hematogenic emboli from a pulmonary lesion [5,13,14]; but the patient in our report had no evidence of leptomeningeal or pulmonary involvement. In patients without history of systemic neoplasia such as the patient in our report, a knowledgeable clinical history and a hands on physical examination coupled with selected laboratory and diagnostic imaging investigations may delineate primary or secondary spinal malignant lesions from other more benign differential diagnostic entities. MRI is useful in determining the extent of CNS involvement which may affect the therapeutic decision making in many patients [12,15]. Pain, weakness, sensory deficits and bowel/bladder dysfunction are the presenting complaints of ISCM. Germ et al reported pain and weakness to be the most frequent. Among the 55 patients reviewed by them, bowel and bladder dysfunctions were unusual early manifestations of intramedullary spinal cord metastasis probably because the time course from the onset of neurologic symptoms to the development of the full blown neurological deficit was short [4]. According to Schiff and O’ Nill , median duration of symptoms at the diagnosis was 28 days (ranged from 3 days to 18 months); median survival was 4 months for patients receiving radiotherapy and 2 months for those not receiving radiotherapy [5]. Their finding was consistent with that of Germ et al who found that more than 80% of patients died within three months after diagnosis of ISCM [4].

The prognosis of a patient who has an intramedullary cord lesion is grave, the treatment is mostly undertaken to relieve pain and to preserve or stabilize neurologic function. Medical and surgical interventions and radiotherapy have controversially been used as a therapeutic modality. The management of patients has evolved greatly over the last decade. Spine surgeons are playing greater role in the management of patients with metastatic disease. With the advent of new surgical strategies many patients may benefit from effective treatment modalities ranging from radical, open excision through minimally invasive surgery such as endoscopy to ultraminimal/noninvasive spinal radiosurgery. Radiotherapy may no longer be considered as the first line therapeutic modality. Making an early diagnosis of ISCM is useful in planning either early or no major intervention. Providing patients with successful palliation and improving their quality of life demand multidisciplinary strategic treatment planning [12,16].

## Conclusion

This report presents the clinical and neuroradiological features of an extremely unusual presentation of renal cell carcinoma as intramedullary spinal cord lesion.

## Abbreviation

MR: Magnetic resonance

## References

[bib-001] Subačiūtė J (2007). The treatment of malignant spinal cord tumours. Acta Medica Lituanica.

[bib-002] Reddy P, Sathyanarayana S, Acharya R, Nanda A (2003). Intramedullary spinal cord metastases: case report and review of literature. J La State Med Soc.

[bib-003] Mut M, Schiff D, Shaffrey ME (2005). Metastasis to nervous system: spinal epidural and intramedullary metastases. J Neurooncol.

[bib-004] Grem JL, Burgess J, Trump DL (1985). Clinical features and natural history of intramedullary spinal cord metastasis. Cancer.

[bib-005] Schiff D, O’Neill BP (1996). Intramedullary spinal cord metastases: clinical features and treatment outcome. Neurology.

[bib-006] Lee SS, Kim MK, Sym SJ, Kim SW, Kim WK, Kim SB, Ahn JH (2007). Intramedullary spinal cord metastases: a single-institution experience. J Neurooncol.

[bib-007] Marquart C, Weckesser M, Schueller P, Hasselblat M, Wassmann H, SchrÖder J (2007). Intramedullary spinal cord metastasis as initial presentation of systemic cancer-report of a rare case. Zentralbl Neurochir.

[bib-008] Grasso G, Meli F, Patti R, Giambartino F, Florena AM, Iacopino DG (2007). Intramedullary spinal cord tumor presenting as the initial manifestation of metastatic colon cancer: case report and review of the literature. Spinal Cord.

[bib-009] Donovan DJ, Freeman JH (2006). Solitary intramedullary spinal cord tumor presenting as the initial manifestation of metastatic renal cell carcinoma: case report. Spine.

[bib-010] Watanabe M, Nomura T, Toh E, Sato M, Mochida J (2006). Intramedullary spinal cord metastasis: a clinical and imaging study of seven patients. J Spinal Disord Tech.

[bib-011] Fakih M, Schiff D, Erlich R, Loqan TF (2001). Intramedullary spinal cord metastasis (ISCM) in renal cell carcinoma: a series of six cases. Ann Oncol.

[bib-012] Jacobs WB, Perrin RG (2001). Evaluation and treatment of spinal metastases: an overview. Neurosurg Focus.

[bib-013] Constans JP, de Divitiis E, Donzelli R, Spaziante R, Meder JF, Haye C (1983). Spinal metastases with neurological manifestations. Review of 600 cases. J Neurosurg.

[bib-014] Costigan DA, Winkelman MD (1985). Intramedullary spinal cord metastasis. A clinicopathological study of 13 cases. J Neurosurg.

[bib-015] Post MJ, Quencer RM, Green BA, Montalvo BM, Tobias JA, Sowers JJ, Levin IH (1987). Intramedullary spinal cord metastases, mainly of nonneurogenic origin. AJR Am J Roentgenol.

[bib-016] Klimo P, Schmidt MH (2004). Surgical management of spinal metastases. Oncologist.

